# 

**Published:** 1918-05-25

**Authors:** 


### CONTENTS.

| | PAGE |
|---|---|
| **A Challenge to the Colleges** ... ... ... ... | 151 |
| **The Cultivation of Legacies: Help the Lawyers to Help the Hospitals** ... ... | 152 |
| **Hospital and Institutional News** ... ... ... | 153 |

The After-care of Discharged Warriors—Sectional Subscriptions in London—A Guide Through the Official Maze—"Sacrilege" at a Military Hospital—The Bradford School Clinic Criticised—The New Call-up and Subscriptions—The Ultimate Test of an Appeal—A "Strike" of Colliery Doctors—A Hospital Secretary's Retirement—Hospital Plans in Acton—A Weak Defence of the Letter System—Rhyl Red Cross Hospital—Stethoscope Boils—The Health of German Women—Hospitals and a Rumour—Lip-reading for Deaf Sailors and Soldiers—Workhouses After the War—Surgery Under the Poor-Law—Tuberculosis More Rife—The New Maternity Bill—A Progressive Disease—Government Blankets for Institutions—Dispensers' Salaries—The Programme for "Baby Week"—The Cost of Municipal Crèches—This Week's Drug Market.

| | |
|---|---|
| **The Great War** ... ... ... ... ... ... | 157 |

VIII. Sanitation.

| | |
|---|---|
| **With the Red Cross in Palestine** ... ... | 158 |
| **The Position of Science in Education** ... ... | 159 |

Report of the Prime Minister's Committee: III. Abolish Present Waste of Intellect and Provide Better System.

| | PAGE |
|---|---|
| **The Case Against the Comforts Depot** ... | 160 |
| **A Ministry of Health** ... ... ... ... | 161 |

The Scheme of the British Medical Association.—II.

| | |
|---|---|
| **Tuberculosis Topics** ... ... ... ... ... | 162 |
| **Fewer Suicides...** ... ... ... ... ... | 163 |

Due to Less Drinking and Increased Employment.

| | |
|---|---|
| **The Economics of Nursing...** ... ... ... | 164 |

A Nurse's Charter of Liberty.

| | |
|---|---|
| **The Matrons' and Sisters' Department** ... | 165 |

The Growth of the Nursing Profession: IX. Some War Thoughts and a New Spirit.
Round the Hospitals.
Programme of College Conference—Nation's Fund: Edinburgh Meeting—A Nurses' Charter of Liberty—Matrons in Cottage Hospitals—V.A.D.s and the Salary Question—A Pension Scheme for District Nurses.

| | |
|---|---|
| **The Nation's Fund for Nurses** ... ... ... | 169 |

Drawing-room Meeting in Edinburgh.

| | |
|---|---|
| **The Editor's Letter-Box** ... ... ... ... | 170 |

Which is the Oldest Hospital?—Changes in the System of Nurse Training at the London Hospital.

| | |
|---|---|
| **Parliament and the Profession** ... ... ... | 170 |
| **Some Problems of To-Day** ... ... ... ... | 171 |
| **Forthcoming Events** ... ... ... ... ... | 172 |
| **Matrons' and Sisters' Appointments** ... .. | 172 |

